# The use of antibiotics in COVID-19 management: a rapid review of national treatment guidelines in 10 African countries

**DOI:** 10.1186/s41182-021-00344-w

**Published:** 2021-06-23

**Authors:** Yusuff Adebayo Adebisi, Nafisat Dasola Jimoh, Isaac Olushola Ogunkola, Theogene Uwizeyimana, Alaka Hassan Olayemi, Nelson Ashinedu Ukor, Don Eliseo Lucero-Prisno

**Affiliations:** 1 Faculty of Pharmacy, University of Ibadan, Ibadan, Nigeria; 2 Medical Research Center, Kateb University, Kabul, Afghanistan; 3 Federal University of Technology Minna, Minna, Nigeria; 4 Department of Public Health, University of Calabar, Calabar, Nigeria; 5 Department of Public Health, Mount Kenya University Rwanda, Kigali, Rwanda; 6 Department of Microbiology, Obafemi Awolowo University, Ile-Ife, Nigeria; 7 Faculty of Pharmacy, University of Port Harcourt, Choba, Nigeria; 8 Department of Global Health and Development, London School of Hygiene and Tropical Medicine, London, UK

**Keywords:** COVID-19, Clinical case management, Antimicrobial resistance, Antibiotic resistance, Pandemic, Africa

## Abstract

Antimicrobial resistance is a hidden threat lurking behind the COVID-19 pandemic which has claimed thousands of lives prior to the emergence of the global outbreak. With a pandemic on the scale of COVID-19, antimicrobial resistance has the potential to become a double-edged sword with the overuse of antibiotics having the potential of taking us back to the pre-antibiotic era. Antimicrobial resistance is majorly attributed to widespread and unnecessary use of antibiotics, among other causes, which has facilitated the emergence and spread of resistant pathogens. Our study aimed to conduct a rapid review of national treatment guidelines for COVID-19 in 10 African countries (Ghana, Kenya, Uganda, Nigeria, South Africa, Zimbabwe, Botswana, Liberia, Ethiopia, and Rwanda) and examined its implication for antimicrobial resistance response on the continent. Our findings revealed that various antibiotics, such as azithromycin, doxycycline, clarithromycin, ceftriaxone, erythromycin, amoxicillin, amoxicillin-clavulanic acid, ampicillin, gentamicin, benzylpenicillin, piperacillin/tazobactam, ciprofloxacin, ceftazidime, cefepime, vancomycin, meropenem, and cefuroxime among others, were recommended for use in the management of COVID-19. This is worrisome in that COVID-19 is a viral disease and only a few COVID-19 patients would have bacterial co-infection. Our study highlighted the need to emphasize prudent and judicious use of antibiotics in the management of COVID-19 in Africa.

To the editor:

COVID-19 continues to threaten health systems globally and African countries are not spared [1, 2]. Prior to the COVID-19 outbreak, antimicrobial resistance (AMR) has been a “hidden” pandemic threatening healthcare delivery worldwide, claiming 700,000 deaths per year [3]. According to the World Health Organization (WHO), AMR occurs when pathogens such as viruses, bacteria, parasites, and fungi undergo changes and no longer respond to treatment making infections difficult to treat, thus increasing the risk of disease spread, poor outcomes, and mortality [4]. In 2019, the WHO also identified AMR as one of the major threats facing healthcare systems [5]. AMR is a growing global health issue to which the present COVID-19 outbreak may contribute [3]. This situation is further complicated with the pressure to repurpose drugs to treat COVID-19, deteriorating economic conditions, and the shifting of resources away from antimicrobial stewardship programs resulting to indiscriminate use of antibiotics in COVID-19 treatment [6]. Presently, the COVID-19 is ruling all aspects of healthcare globally, including health systems response to antimicrobial resistance and the impact will persist for a while, even after the pandemic. With the alarming increase in antibiotic resistance cases and the fact that there are few new antimicrobial agents in the pipeline, it is important to monitor the epidemiology of pathogens to make informed treatment decisions.

In this paper, we conducted a rapid review of national treatment guidelines for COVID-19 in 10 African countries and examined its implication for antimicrobial resistance response on the continent. The 10 African countries include Ghana, Kenya, Uganda, Nigeria, South Africa, Zimbabwe, Botswana, Liberia, Ethiopia, and Rwanda. The countries were selected at random with no predetermined criterion. An online search was conducted to retrieve the national treatment guidelines for the management of COVID-19 in these countries through the government/ministry of health websites. The report guidelines were reviewed to understand the use of antibiotics in the management of COVID-19, i.e., which antibiotics and in what scenario they were recommended.

In Table 1, we summarize our findings on the use of antibiotics in the management of COVID-19. Our findings revealed that various antibiotics such as azithromycin, doxycycline, clarithromycin, ceftriaxone, amoxicillin, amoxicillin-clavulanic acid, ampicillin, gentamicin, erythromycin, benzylpenicillin, piperacillin/tazobactam, ciprofloxacin, ceftazidime, cefepime, vancomycin, meropenem, and cefuroxime were recommended for use in the management of COVID-19, i.e., asymptomatic, mild, moderate, and severe COVID-19 with/without complications. Most of the guidelines recommended directed and empiric therapy with antibiotics. The WHO recommended that antibiotic therapy or prophylaxis should not be used in patients with mild/moderate COVID-19 unless it is justifiable [7]. Interestingly, according to our findings, some countries still recommended the use of antibiotics in the management of mild COVID-19. Most antibiotics recommended across the African countries were from the “watch” (antibiotics that have higher resistance potential) and “reserve” (antibiotics and antibiotic classes that should be reserved for treatment of confirmed or suspected infections due to multi-drug-resistant organisms) categories of WHO AWaRe classification, which may be further adding “fuel to the fire” of the already fearsome antimicrobial resistance situation. Our study reiterates the need to go revisit fundamentals of diagnostic stewardship and practice culture-directed therapy using narrow-spectrum antibiotics, from the “access” category of AWaRe classification which has lower resistance potential than antibiotics in the other groups.

**Table 1:** The use of antibiotics in COVID-19 management in 10 African countries

Country	List of antibiotics recommended in the guideline	Scenario for recommendation	Compliance with WHO guideline	Guideline references (accessed 4 June 2021)
Ghana	Azithromycin, doxycycline	Recommended for use in the management of confirmed cases (with asymptomatic, mild, or moderate symptoms)	-WHO does not recommend antibiotic use in suspected/mild/moderate COVID-19. -WHO does not recommend azithromycin with/without hydroxychloroquine in the management of COVID-19. -WHO does not encourage the use of broad-spectrum antibiotics for COVID-19 especially those on the Watch and Reserve List.	https://www.moh.gov.gh/wp-content/uploads/2016/02/COVID-19-STG-JUNE-2020-1.pdf
Kenya	Amoxicillin, amoxicillin-clavulanic acid, erythromycin, azithromycin, clarithromycin	Recommended for use in the management of severe COVID-19 and sepsis. Empirical use of antimicrobials for all severe acute respiratory infections and should be de-escalated on the basis of microbiology results and clinical judgment	-WHO does not recommend azithromycin with/without hydroxychloroquine in the management of COVID-19 - WHO does not encourage the use of broad-spectrum antibiotics for COVID-19 especially those on the Watch and Reserve List.	https://kma.co.ke/Documents/Case%20management%20protocol.pdf
Uganda	Azithromycin and amoxicillin (moderate COVID-19); ceftriaxone, ampicillin, gentamicin, benzylpenicillin, and azithromycin (severe COVID-19 pneumonia); and azithromycin, piperacillin/tazobactam (critically ill COVID-19 patient)	Empiric use of antibiotics is recommended for sepsis in COVID-19 patient as well as in moderate, severe, and critically ill COVID-19 patient and de-escalated on the basis of microbiology results and clinical judgment	-WHO does not recommend antibiotic use in mild/moderate COVID-19. -WHO does not encourage the use of broad-spectrum antibiotics for COVID-19 especially those on the Watch and Reserve List.	https://covidlawlab.org/wp-content/uploads/2020/06/National-Guidelines-for-Clinical-Management-of-Covid-19.pdf
Nigeria	No specific antibiotic was stated in the treatment guideline for COVID-19. However, broad-spectrum antibiotics based on local epidemiology were recommended for some cases	Prophylactic/empiric use of antibiotics is not recommended in asymptomatic and mild COVID-19 cases. For severe COVID-19 cases, the choice of antibiotics should be based on the clinical diagnosis, local epidemiology, and antibiotic susceptibility	The country’s guideline complies with WHO treatment guidelines for COVID-19 and does not list any specific antibiotic for use in COVID-19 management.	https://covid19.ncdc.gov.ng/media/files/National_Interim_Guidelines_for_Clinical_Management_of_COVID-19_v3.pdf
South Africa	Ceftriaxone and azithromycin	Empirical use of antibiotics is recommended for co-infections such as conventional community-acquired pneumonia or atypical pneumonia	WHO does not encourage the use of broad-spectrum antibiotics for COVID-19 especially those on the Watch and Reserve List.	https://www.nicd.ac.za/wp-content/uploads/2020/03/Clinical-Management-of-COVID-19-disease_Version-3_27March2020.pdf
Zimbabwe	Ceftriaxone and azithromycin	Recommend that antimicrobial therapy should not be delayed just to collect blood culture. Empiric antibiotics are recommended	WHO does not encourage the use of broad-spectrum antibiotics for COVID-19 especially those on the Watch and Reserve List.	https://cquin.icap.columbia.edu/wp-content/uploads/2020/04/ZIMBABWE_COVID-19-CLINICAL-GUIDELINES-APRIL-2020.pdf
Botswana	Amoxicillin-clavulanic acid and azithromycin (suspected/confirmed COVID-19 cases)	If clinical suspicion for co-infection exists, consider empirical antimicrobials to treat co-pathogens causing the syndrome	-WHO does not encourage the use of broad-spectrum antibiotics for COVID-19 especially those on the Watch and Reserve List. -WHO does not recommend antibiotic use in mild/moderate/suspected COVID-19. -WHO does not recommend azithromycin alone or with/without hydroxychloroquine in the management of COVID-19.	https://covid19portal.gov.bw/sites/default/files/2020-05/Interim-COVID-19-Clinical-Management-Guideline-Botswana.pdf
Liberia	Amoxicillin-clavulanic acid, azithromycin, amoxicillin (moderate COVID-19) and amoxicillin-clavulanic, gentamicin, doxycycline acid, azithromycin, and ampicillin (severe COVID-19). Fluoroquinolones, e.g., ciprofloxacin with or without metronidazole in COVID-19-related symptoms.	Empiric use of antibiotic (broad spectrum) is recommended for severe and mild case. For COVID-19-associated sepsis, laboratory findings such as blood cultures, sputum culture, chest X-ray, examination of line sites, etc. are recommended to guide antibiotic selection. Antibiotics are also recommended for sore throat in mild/moderate COVID-19, as well as cough, and diarrhea.	WHO does not encourage the use of broad-spectrum antibiotics for COVID-19 especially those on the Watch and Reserve List. -WHO does not recommend antibiotic use in mild/moderate/suspected COVID-19. -WHO does not recommend azithromycin or with/without hydroxychloroquine in the management of COVID-19.	http://moh.gov.lr/wp-content/uploads/Interim_Guidance_for_care_of_Pts_with_Covid_19_in_Liberia.pdf
Ethiopia	Amoxicillin-clavulanic acid or amoxicillin (moderate COVID-19) and ceftazidime/cefepime and/or vancomycin or meropenem (other carbapenems) ± vancomycin (severe/critical COVID-19). Recommended antibiotics in pediatrics also include gentamicin, ampicillin, ceftriaxone/cefotaxime, azithromycin, and meropenem	Antibiotics (preferably broad spectrum) are recommended for empiric use based on physician judgment after taking a sample for blood culture, in severe COVID-19 cases	WHO does not encourage the use of broad-spectrum antibiotics for COVID-19 especially those on the Watch and Reserve List. -WHO does not recommend antibiotic use in mild/moderate/suspected COVID-19. -WHO does not recommend azithromycin or with/without hydroxychloroquine in the management of COVID-19.	https://www.moh.gov.et/ejcc/sites/default/files/2020-09/National%20Comprehensive%20COVID%2019%20Clinical%20Management%20Handbook%20Second%20Edition.pdf
Rwanda	Doxycycline, amoxicillin, amoxicillin-clavulanic acid (moderate and mild COVID-19), clarithromycin, amoxicillin-clavulanic acid, cefuroxime, ceftriaxone, or levofloxacin [if allergy to penicillin] (severe and critical COVID-19)	Recommend antibiotics for highly suspected pneumonia based on clinical signs in moderate/mild COVID-19, prevention of secondary bacterial infection, and ventilator-associated pneumonia in severe/critically ill COVID-19 patient	-WHO does not recommend the use of antibiotics in mild/moderate COVID-19.	https://www.rbc.gov.rw/fileadmin/user_upload/guide/Guidelines/COVID-19%20Clinical%20Managment%20guidelines.pdf

Empirical use of antibiotics is a risk factor for development of resistance [8], and in the case of COVID-19, this situation in resource-limited settings remains worrisome because of the weak laboratory systems, ineffective antimicrobial stewardship, lack of human and financial resources, prescribers’ opposition, limited access to medicines, lack of awareness and absence of antimicrobial stewardship committees, concerns regarding fake and counterfeit antibiotics, limited hospital infection prevention program infrastructure, and lack of effective antibiotic policy among others [6]. Our findings also show that broad-spectrum antibiotics were the most recommended antibiotics with the drawback of selection for resistance [9]. The WHO has also warned against any indiscriminate use of (broad-spectrum) antibiotics in the management of COVID-19 [7]. Our review also revealed that the national treatment guideline of Liberia recommended the use of antibiotics in sore throat, diarrhea, and cough that are associated with COVID-19 symptoms. This highlights the need to ensure prudent use of antibiotics in COVID-19, being a viral disease.

Various studies have also shown that most bacterial pneumonias that are diagnosed early in COVID-19 patients can be safely and effectively treated with antibiotics, and broad-spectrum antibiotics are widely used [10–12]. A recent review article that pooled data from 19 studies (2834 patients) revealed that the mean rate of antibiotic use in COVID-19 management is 74.0% and only 17.6% of patients had secondary infections [13]. Another study conducted in South Africa revealed that bacterial co-infection is rare at the time of intensive care unit admission with COVID-19 [14]. Another meta-analysis revealed that only 7.0% of hospitalized COVID-19 patients had a bacterial co-infection [15]. A recent multi-center study showed that only 86 out of 905 (9.5%) confirmed COVID-19 patients were clinically diagnosed with bacterial co-infection [16]. This implies that only a few COVID-19 patients would need antibiotics for possible bacterial pneumonia and other superimposed/co-infections [17].

For patients who are critically ill and hospitalized, the diagnosis of a potential bacterial co-infection is uncertain; physicians tend to use broad-spectrum antibiotics to manage such patients [18]. An increase in usage of broad-spectrum antibiotics from the “watch” and “reserve” categories will not only make the agents ineffective but will also create highly drug-resistant bugs which may become clinicians’ nightmare. This is a major threat to antimicrobial stewardship. For instance, an increase in the use of azithromycin, a broad-spectrum macrolide antibiotic, has been documented amid the pandemic in many African countries [19, 20], usually with hydroxychloroquine in the management of COVID-19. Evidence has also shown that routine use of azithromycin for reducing time to recovery or risk of hospitalization for people with suspected COVID-19 in the community has been documented to offer no benefit [21–23]. In summary, antibiotics need to be used with care and should be withheld unless it is confirmed that the patient truly needs them. While lack of access to antibiotics could be dangerous in the same vein as its misuse, it is of importance to ensure that these life-saving agents are preserved and used with utmost care [18].

African countries are vulnerable to the looming threat of the antimicrobial resistance. This is worrisome because pathogens that cause resistant infections thrive in hospitals and medical facilities, putting all patients at risk, irrespective of the severity of their medical conditions. The situation is further catalyzed in Africa by unsanitary conditions, high burden of infectious diseases, inadequate access to clean water, conflicts, poor coverage of vaccination program, and growing numbers of immunosuppressed people, such as those living with HIV, which facilitate both the evolution and emergence of resistant organisms and their sporadic spread in the community. In addition, judicious empirical use of antibiotics in Africa will be challenging because of the lack of widespread data on antimicrobial resistance and ease of purchase of antibiotics over the counter without a prescription. Many African countries are also yet to align with the international efforts to fight the increasing antibiotic resistance in that only seven African countries have developed the national action plan on antimicrobial resistance [24]. Our review highlighted the need to emphasize prudent use of antibiotics in the management of COVID-19 in Africa by strengthening antimicrobial stewardship programs on the continent.

The COVID-19 pandemic reveals that we remain susceptible to infections for which we have no specific treatment options [25, 26]. This is a wakeup call to African countries to ensure investment in antimicrobial stewardship in order to optimize antibiotic use by ensuring that the appropriate antibiotic is administered at the right dose, for the right duration, and in a way that ensures the maximum outcome and reduces any untoward effect and development of resistance. Diagnostic precision and addressing diagnostic insufficiency are also crucial in modifying the current approach of widespread empirical antibiotic use in the management of COVID-19. We also call on national health authorities in African countries to ensure their treatment guidelines for COVID-19 do not encourage the injudicious use of antibiotics. All countries should also implement measures to track the use of antibiotics and comply with the WHO’s guideline to promote antibiotic stewardship amid the COVID-19 pandemic. Countries should also invest in continuous training of their healthcare workers on antimicrobial stewardship.

## Data Availability

Data sharing is not applicable to this article as no datasets were generated or analyzed.

## Abbreviations

COVID-19: Coronavirus disease
AMR: Antimicrobial resistance
WHO: World Health Organization
